# Governing multisectoral action for health in low- and middle-income countries

**DOI:** 10.1371/journal.pmed.1002285

**Published:** 2017-04-25

**Authors:** Kumanan Rasanathan, Sara Bennett, Vincent Atkins, Robert Beschel, Gabriel Carrasquilla, Jodi Charles, Rajib Dasgupta, Kirk Emerson, Douglas Glandon, Churnrurtai Kanchanachitra, Pete Kingsley, Don Matheson, Rees Murithi Mbabu, Charles Mwansambo, Michael Myers, Jeremias Paul, Thulisile Radebe, James Smith, Orielle Solar, Agnès Soucat, Aloysius Ssennyonjo, Matthias Wismar, Shehla Zaidi

**Affiliations:** 1 UNICEF, New York, New York, United States of America; 2 Johns Hopkins University Bloomberg School of Public Health, Baltimore, Maryland, United States of America; 3 Caribbean Community Secretariat, Saint Michael, Barbados; 4 World Bank, Washington, D.C., United States of America; 5 Fundación Santa Fe de Bogotá, Bogotá, Colombia; 6 U.S. Agency for International Development (USAID), Washington, D.C., United States of America; 7 Jawaharlal Nehru Medical College, New Delhi, India; 8 University of Arizona, Tucson, Arizona, United States of America; 9 Mahidol University, Nakhon Pathom, Thailand; 10 University of Edinburgh, Edinburgh, United Kingdom; 11 Independent consultant, Brisbane, Australia; 12 Ministry of Agriculture, Livestock and Fisheries, Nairobi, Kenya; 13 Ministry of Health, Lilongwe, Malawi; 14 The Rockefeller Foundation, New York, New York, United States of America; 15 World Health Organization (WHO), Geneva, Switzerland; 16 Ministry of Public Service and Administration, Pretoria, South Africa; 17 Facultad Latinoamericana de Ciencias Sociales (FLACSO), Santiago, Chile; 18 Makerere University, Kampala, Uganda; 19 World Health Organization (WHO), Brussels, Belgium; 20 Aga Khan University, Karachi, Pakistan

## Abstract

Kumanan Rasanathan and colleagues argue that the potential of multisectoral collaboration for improving health remains untapped in many low- and middle-income countries.

**Summary points**

- The focus of the health sector in most countries remains almost exclusively on health care services, and the potential of multisectoral collaboration remains untapped in many low- and middle-income countries.
- Different sectors have different contributions to make towards solving specific health problems. In each case, the profile, interests, incentives, and relationships of key individuals and sectors must be mapped and analysed to inform the design of approaches and systems to tackle a shared problem.
- Collaborative and distributed leadership is key for effective governance of multisectoral action, with a need to build leadership capacity across sectors and levels of government and cultivate champions in different sectors who can agree on common objectives.
- Important ways forward to support countries to take a multisectoral approach for health include ensuring that the universal health coverage agenda addresses the capacity of the health sector to work with other sectors, learning from multisectoral efforts that do not involve the health sector, improving the capacity of global institutions to support countries in undertaking multisectoral action, and developing a clear implementation research agenda for multisectoral action for health.

## Introduction

The 2030 Agenda for Sustainable Development adopted by countries at the United Nations in 2015 sets forth a comprehensive vision of development with 17 Sustainable Development Goals (SDGs) and 169 targets across all aspects of society [1]. The 2030 Agenda document is ambitious and explicit about the need for integrated and sustained action across society to address complex challenges such as ending extreme poverty, reducing widening economic inequality, tackling climate change, and reducing and preventing conflict. These issues are beyond the remit and resources of a single thematic sector (such as health, finance, agriculture, or education) and instead require coordinated multisectoral action.

In the lead-up to Agenda 2030, the global health community argued that health is a prerequisite, contributor, and indicator for sustainable development [2]. Unlike the more selective Millennium Development Goals, in which three out of eight goals were specifically health focused, in the SDGs there is only one specific health goal (namely, SDG 3). At the same time, there are targets that are essential to health improvement across the other 16 SDGs. Most of the targets under the health SDG (such as ending preventable child deaths or ending the HIV epidemic) are unachievable by the health sector alone and are dependent on achievement of SDG targets under other goals. The SDGs thus refocus attention on how multisectoral action can be implemented to improve health outcomes and how the health sector can cooperate with other sectors to achieve the array of targets in the SDGs, not just those contained in SDG 3. An emphasis on multisectoral action also provides a way to transcend concerns among some in the health community that health is underrepresented in the SDG agenda or the countervailing critique that health was overrepresented in the MDGs.

The call for multisectoral action for health is not new. In advocating primary health care, the Declaration of Alma Ata in 1978 stated upfront that realizing the right to health is a “social goal whose realization requires the action of many other social and economic sectors in addition to the health sector” [3]. Despite a number of important initiatives promoting multisectoral action since then [4–9] and many examples of success in improving health, the focus of the health sector in most countries remains almost exclusively on health care services, and the potential of multisectoral collaboration remains untapped, particularly in many low- and middle-income countries.

At its core, multisectoral collaboration concerns governance—“the complex mechanisms, processes, relationships and institutions through which citizens and groups articulate their interests, exercise their rights and obligations, and mediate their differences” [10]. Effective governance is key to the development of shared policy visions and, even more critically, the effective implementation of programmes and policies that require coordination across different sectoral agencies and different levels of government [11]. It is surprising therefore that prior work has not drawn fully on learning from political economy, public administration, or public management, which have much to offer on how to address head-on the political challenges of bringing together sectors with differing, and sometimes conflicting, mandates, funding streams, interests, incentives, languages, and disciplinary cultures—particularly in low- and middle-income countries. The exception to this omission lies with the body of work in recent years examining the “Health in All Policies” approach [12], but the bulk of these experiences have been from a small number of high-income countries, and the health sector has mostly retained the dominant role. Too often, the health sector approaches multisectorality in a siloed fashion, examining it through the lens of one particular illness or condition. The health sector is also frequently guilty of “health imperialism” [13], viewing other sectors as serving its needs without considering the interests of those sectors to which it might contribute.

The literature from public administration on “collaborative governance” reflects the increasing application of multisectoral approaches in a variety of public policy fields, such as environmental protection, emergency management, land use planning, and education policy [14,15]. This scholarship provides useful theories and lessons that are transferable to the challenges of implementing successful multisectoral action for health. Key findings include the importance of the institutional environment [16], leadership [17], trust [18], analysis of networks [19], agreement on problem definition [20], and fostering the ability to manage conflicts [21]. An emerging body of work also considers the quality of collaboration dynamics in a given context and its impact on multisectoral action [22]. Literature specific to low- and middle-income countries has made less inroads in understanding collaborative governance, but key conclusions regarding strengthening public sector capacity tend to emphasize similar factors, notably communication networks, leadership (including strength of domestic ownership of issues), trust, and institutional environments that support transparency and accountability and create incentives for performance [23–25].

Given this context and the opportunities presented by the SDGs, and cognizant of these gaps, we convened as a diverse group from different countries, sectors, and disciplines at the Rockefeller Foundation’s Bellagio Center in June 2016 to reflect on country experiences of multisectoral action in low- and middle-income countries. In particular, we sought to identify political economy and governance challenges, as well as potential solutions, to inform how multisectoral action for health should be pursued to realize the SDGs. We aimed to consider multisectoral action in terms of how the health targets in SDG 3 can be achieved through the contributions of different sectors but also to reflect on how the broader SDG agenda for human well-being requires coordinated actions across society, with the health sector, and health as an outcome, being only one ingredient. Here we present key insights that we collectively identified from our discussions, which consisted of three days of presentations of country case studies and analytical frameworks, plenaries, and small-group work. Five background papers were prepared for the meeting, and fully developed versions of these manuscripts are planned to be published elsewhere.

## Key considerations and lessons for successful governance of multisectoral action

Examining experiences (three examples are presented in Box 1) of multisectoral collaborations for health from Cambodia, Chile, Colombia, India, Kenya, Malawi, Pakistan, the Philippines, Thailand, Uganda, the Caribbean, the Pacific, and the European region, we identified five key considerations and lessons for successful governance of multisectoral action.

**Box 1. Examples of how governance affects multisectoral action for health**

**Coordinating sleeping sickness control in Uganda**

Trypanosomiasis, or “sleeping sickness,” is a vector-borne disease that has proved to be an ongoing challenge in Uganda. Tackling it requires particularly close collaboration between the health and agriculture sectors. Different actors in Uganda frame sleeping sickness in different ways—as a possible future threat, an ongoing emergency, or a disease ripe for elimination—making harmonisation difficult. A national coordinating body for trypanosomiasis, founded in 1992, pioneered multisectoral and interagency collaboration, albeit with limited ability to shape externally funded projects. However, political reforms such as privatization and decentralization have diminished local capacity to control the disease. Global policy has also had mixed consequences: the “One Health” agenda is broadly supportive of multisectoral collaboration, yet research projects and interventions often reflect global interests rather than local priorities [26]. Ongoing efforts for trypanosomiasis control therefore require greater attention to these political economy issues, particularly around framing of the issue and the impact of broader political reform on multisectoral collaboration.

**Comparison of multisectoral action on undernutrition and early childhood development in Pakistan**

Pakistan has undertaken specific multisectoral efforts on two priorities for child health and well-being over the last two decades: undernutrition and early childhood development. It is instructive to compare their relative trajectories. Nesting multisectoral initiatives within existing government structures (as occurred more for efforts on undernutrition) has provided better sustainability compared to special programmes (as was seen more for early childhood development), although at the cost of slower implementation. For undernutrition, powerful advocacy coalitions and a clear actionable agenda have also emerged as enablers for uptake of policy agendas across sectors [27]. Weak capacities for planning, financing, and monitoring were common challenges impeding multisectoral implementation for both issues. Given the continuing salience of these two issues for child health, new efforts for early childhood development should learn from the experience with undernutrition, and strategies for both issues need to pay more attention to the institutionalization of related programmes in existing structures.

**Enacting and implementing “sin taxes” in the Philippines**

In 2012, the Philippines enacted and implemented legislation for “sin taxes” for alcohol and tobacco consumption after a long battle [28]. The health benefits, strongly supported by evidence from other countries, were not sufficient to win political support to pass the legislation. Instead, the turning point was when the reform was framed as a health measure with additional revenues from higher sin taxes earmarked to finance the politically popular universal health care programme. Champions from the Ministries of Health and Finance worked together with a civil society coalition to enlist the support of Congress and other political leaders. This example of a successful multisectoral effort between finance and heath sectors for “sin taxes” has since been replicated in other jurisdictions. It is unclear, however, whether this experience will lead to sustained improvements in collaboration between these two sectors towards improved health outcomes.

First, the most important barriers to multisectoral action, including to implementation, are political, not technical, yet these aspects are often neglected. For example, how an issue is framed—whether it be in terms of development, equity, economic, or health gains—and the extent to which this resonates with high-level political agendas are crucial to achieving buy-in from different sectors. Similarly, navigating tacit institutional hierarchies, and where health ministries fit within them, may be key to allocating leadership roles on a health issue. For multisectoral collaboration to be effective, a detailed understanding of the political ecosystem is required, including the historical context and how this influences different stakeholders’ interests and approaches to the problem.

Second, there are diverse types of multisectoral action (Box 2), from light-touch coordination across sectors to collaborative problem solving for deeply rooted social problems. Across these forms, different actors may be involved, and contexts in terms of history, institutional capabilities, and accountabilities vary enormously. Accordingly, the requirements for governance of multisectoral action also vary. While it can be useful to understand the type of multisectoral action being pursued, this needs to be accompanied by mapping of the profile, interests, incentives, and relationships of key individuals and sectors. The design of approaches and systems for multisectoral action must be informed by analysis of these specific circumstances.

**Box 2. What is multisectoral action for health?**

“Multisectoral action for health” encompasses all activities involving nonhealth sectors that can potentially improve health. In our meeting, we adapted an existing typology [29] to differentiate four broad types of multisectoral action, noting that they overlap:

1. Health sector as a minimal actor in contexts where other individual sectors undertake their core business (such as ensuring children attend school and learn well for the education sector or access to clean power for the energy sector) and have spillover effects for health;
2. Health sector as a supporting actor, e.g., for cross sectoral policies to address structural forces and social norms that affect all of society, including those that drive disparities;
3. Health sector as a bilateral or trilateral partner, for example, in contexts where collaboration is required between two or more sectors to produce joint or “co-benefits” and to maximise health benefits (such as the use of cleaner stoves to reduce indoor air pollution, sexuality education in schools, or tobacco taxation to improve both health and revenues);
4. Health sector as a lead actor, e.g., in contexts where collaboration with other sectors is essential for the health sector to deliver its core mandate in delivering health services (such as ensuring adequate water and energy supplies to health facilities or road infrastructure for access).

Third, governing implementation processes for multisectoral action is particularly challenging and requires explicit attention up front. Accountability, transparency, and trust help drive multisectoral action but can be elusive. There are differing organizational and disciplinary cultures between and within sectors, creating difficulties in aligning monitoring indicators and sharing datasets. Assessing and attributing each sector’s contribution is also challenging. Innovation, adaptation, and flexibility are required in terms of political, financial, and administrative accountability to strengthen the governance of multisectoral implementation efforts.

Fourth, collaborative and distributed leadership is key for effective governance of multisectoral action. While there are examples of effective multisectoral efforts due to a single strong leader, dictatorial approaches are not generally the answer, and instead, there is a need to build leadership capacity across sectors and levels of government (particularly as multisectoral action may be easier at decentralized levels) and cultivate champions in different sectors who can agree on common objectives. “Strong man” leadership is rarely fit for purpose; instead, health-sector leadership development should prioritize competencies such as negotiation, flexibility and learning, communication, and relationship building, underpinned by high standards of ethics and integrity. Command- and control-type structures may be effective in emergency situations, but even these are usually affected by limits on information sharing and depend on strong pre-existing relationships and good coordination between sectors and levels of government. Multisectoral governance for health needs to engage nongovernmental stakeholders, including civil society (organized groups and citizens), academia, the private sector, and external funders, with clarity regarding their respective roles. Multisectoral efforts are more likely to succeed if they are institutionalized in existing structures and not championed by a single group or individual. In general, the challenges they tackle are ongoing, so efforts need to be sustained, which is more likely if they are not dependent on a single personality or group or driven by novel structures outside of mainstream systems.

Fifth, there is a particular imperative to cultivate a culture of mutual learning among the divergent stakeholders involved. The innate uncertainties and knowledge gaps with regard to multisectoral action result in a heightened need to course correct as implementation proceeds. This includes building indicators and methods for monitoring and evaluation and supporting relevant research. The causes of many multisectoral problems (such as obesity or emerging zoonoses) are complex, interrelated, and adaptive. Developing shared mental models (such as theories of change or logic or outcome models) of the problem and its solutions can both help get stakeholders on the same page and guide monitoring and evaluation using both quantitative and qualitative methodologies.

## What is required to support countries to govern multisectoral action for health in the SDG era?

We believe that there has been insufficient attention to the political and governance aspects of multisectoral action for health, particularly in low- and middle-income countries. A focus on political economy and governance (beyond the governance of the health sector) provides a new lens to understand why multisectoral action is underutilized or often unsuccessful. Thus, we see the discussions at our Bellagio meeting as an exploratory exercise opening up a field that warrants greater attention and sharing of experiences.

The SDGs provide an important opportunity to more fully realize the potential of multisectoral action for health, but their rollout will not automatically lead to a more collaborative approach, especially as despite the vision of Agenda 2030, the SDGs themselves and their indicators are mostly structured according to sectoral interests. Moving forward will require both addressing knowledge and capacity gaps about multisectoral action within countries and global institutions and, even more critically, changing the paradigm of how health and other social goals are approached. In this respect, while structures and methods for multisectoral action (such as pooling human, material, and financial resources or directly incentivising multisectoral collaboration) are important and specific investments are required to build trust and capacities (including for strategic communication, conflict management, and forming common objectives), policy makers in national and global institutions should give more emphasis to understanding and acting on the political and governance factors highlighted above.

Further efforts could usefully concentrate on a few key directions, identifying opportunities to apply and build existing knowledge on multisectoral action, such as the Health in All Policies and public administrations discourses. For example, the global movement towards universal health coverage has to date paid limited attention to the health sector’s role in working with other sectors. This is despite the goal of universal health coverage itself being dependent on such collaboration—for direct inputs into the delivery of health services (such as roads, energy, information and communications technology, water and sanitation, and training of the health workforce) and for implementing key public health policies (such as tobacco control, road traffic injury prevention, and environmental health). This gap, and its governance implications, should be addressed at both national and global levels under the umbrella of universal health coverage. Partly to address this, we, along with others, will convene a multisectoral working group under the rebranded International Health Partnership for Universal Health Coverage 2030 (UHC2030) partnership, aiming to spark greater attention in building the capacity of the health sector to work with other sectors effectively [30].

There is also much to be learned from the governance of multisectoral experiences that do not involve the health sector, including national level multisectoral planning and coordination structures. The need for multisectoral collaboration is obviously not unique to health goals, yet there has been limited learning from multisectoral efforts towards other sectoral goals. The complex governance arrangements employed successfully in relation to the environment and gender equality, for example, could be examined further to guide health efforts. Given that most of the SDGs require multisectoral action, there is also scope for a learning agenda to mutually reinforce the efforts of countries towards the broad SDG vision. Stewardship for this learning agenda should be taken up by national academic institutions and think tanks, as well as national ministries of health and regional and international organizations.

Indeed, international organizations can play a greater role in providing support for multisectoral governance and addressing the politics of multisectoral collaboration, but first, they need to examine their own internal challenges in working multisectorally and their tendency to foster their own silos. Many global organizations operate explicitly within a single sector or with an exclusive relationship to single ministries. And even multisectoral organizations often struggle to leverage this strength beyond their technical silos. Sectoral specialization, as within national governments, can increase efficiency, and the desire is not for every agency to try to do everything. But the SDG era calls for greater collaboration between different sectoral groups within agencies and between different specialized agencies, particularly at the country level, with simplified and improved governance arrangements in the global system.

Finally, there is a need for the development of a clear implementation research agenda on the governance of multisectoral action, which could provide a rallying point for a community of learning and practice (including assembling and sharing case studies) and should be explicitly supported by global institutions. In order to strengthen national capacity for multisectoral collaboration, research should investigate questions such as the following: How does organizational structure and culture support (or undermine) collaboration? What types of incentives and constraints do public sector officials experience with regard to multisectoral collaboration for health, and what skills do they need to take advantage of collaborative opportunities? How can enabling environments and broader government systems be cultivated to support and sustain multisectoral collaboration? Underpinning each of these questions are political economy perspectives investigating how political institutions, economic systems, and individuals interact to shape multisectoral governance for health.

## Abbreviations

SDG: Sustainable Development Goal
UHC2030: International Health Partnership for Universal Health Coverage 2030
